# Spatial and Temporal Arrangement of Recurrent Inhibition in the Primate Upper Limb

**DOI:** 10.1523/JNEUROSCI.1589-20.2020

**Published:** 2021-02-17

**Authors:** Steve A. Edgley, Elizabeth R. Williams, Stuart N. Baker

**Affiliations:** ^1^Department of Physiology, Development and Neuroscience, Cambridge University, Cambridge CB2 3DY, United Kingdom; ^2^Medical School, Newcastle University, Newcastle upon Tyne NE2 4HH, United Kingdom

**Keywords:** primate, recurrent inhibition, Renshaw cells

## Abstract

Renshaw cells mediate recurrent inhibition between motoneurons within the spinal cord. The function of this circuit is not clear; we previously suggested based on computational modeling that it may cancel oscillations in muscle activity around 10 Hz, thereby reducing physiological tremor. Such tremor is especially problematic for dexterous hand movements, yet knowledge of recurrent inhibitory function is sparse for the control of the primate upper limb, where no direct measurements have been made to date. In this study, we made intracellular penetrations into 89 motoneurons in the cervical enlargement of four terminally anesthetized female macaque monkeys, and recorded recurrent IPSPs in response to antidromic stimulation of motor axons. Recurrent inhibition was strongest to motoneurons innervating shoulder muscles and elbow extensors, weak to wrist and digit extensors, and almost absent to the intrinsic muscles of the hand. Recurrent inhibitory connections often spanned joints, for example from motoneurons innervating wrist and digit muscles to those controlling the shoulder and elbow. Wrist and digit flexor motoneurons sometimes inhibited the corresponding extensors, and vice versa. This complex connectivity presumably reflects the flexible usage of the primate upper limb. Using trains of stimuli to motor nerves timed as a Poisson process and coherence analysis, we also examined the temporal properties of recurrent inhibition. The recurrent feedback loop effectively carried frequencies up to 100 Hz, with a coherence peak around 20 Hz. The coherence phase validated predictions from our previous computational model, supporting the idea that recurrent inhibition may function to reduce tremor.

**SIGNIFICANCE STATEMENT** We present the first direct measurements of recurrent inhibition in primate upper limb motoneurons, revealing that it is more flexibly organized than previous observations in cat. Recurrent inhibitory connections were relatively common between motoneurons controlling muscles that act at different joints, and between flexors and extensors. As in the cat, connections were minimal for motoneurons innervating the most distal intrinsic hand muscles. Empirical data are consistent with previous modeling: temporal properties of the recurrent inhibitory feedback loop are compatible with a role in reducing physiological tremor by suppressing oscillations around 10 Hz.

## Introduction

The activation of Renshaw cells by intraspinal branches of motoneuron axons, and the subsequent inhibition of motoneurons by Renshaw cells, have been a well-established feature of the spinal circuitry for almost 70 years (Eccles et al., 1961; Alvarez and Fyffe, 2007). The precise functions of this recurrent inhibition are less clearly established. Leading hypotheses are that recurrent inhibition performs a filtering function to control the timing of motoneuron spikes, limiting synchrony and enabling smooth force generation (Windhorst and Kokkoroyiannis, 1992; Maltenfort et al., 1998; Williams and Baker, 2009) or that it controls the gain of the motoneuron pool (Hultborn and Pierrot-Deseilligny, 1979; Hultborn et al., 1979).

Given that the neural populations involved cannot be measured experimentally simultaneously and in action, the problem has been previously approached through computational modeling of the system. The results of models are conflicting; some agree with the view that recurrent inhibition decreases synchronous motoneuron firing (Maltenfort et al., 1998), but others find increased synchrony (Uchiyama and Windhorst, 2007). In a recent biophysically-based model (Williams and Baker, 2009), recurrent inhibition did reduce the tendency to synchronous spiking among motoneurons at 10 Hz, but had the converse effect at 30 Hz. Experimental data would clearly be valuable to assess the veracity of the results of these models.

Early experimental studies on this circuit focused on recurrent inhibition in the cat hindlimb, which plays an obligatory role in locomotion and other stereotyped behaviors such as postural set and scratching. The distribution of recurrent inhibition has also been described in the cat forelimb, and generally resembles that in the hindlimb (Illert and Kümmel, 1999). In this study, we report on recurrent inhibition between upper limb motoneurons in non-human primates, which have a major role in manipulation and grasping, functions that involve more flexible and variable patterns of muscle activity, and have greater reliance on corticospinal control. Our experiments had two objectives.

First, the distribution of recurrent inhibition has not been directly studied in primate upper limb motoneurons to date. Indirect studies in man, primarily through conditioning of stretch reflexes, report patterns of recurrent inhibition resembling those seen in cats (Illert and Kümmel, 1999; Katz and Pierrot-Deseilligny, 1999), but the necessarily indirect nature of these measures can lead to ambiguities in interpretation (Özyurt et al., 2019). Given the great flexibility and dexterity of the primate arm compared with the limbs of the cat, with augmented motor cortical control, it is important to know whether the organization of recurrent inhibition follows the principles first elucidated in cat hindlimb.

Second, we have examined temporal characteristics of the recurrent inhibitory pathway directly by delivering trains of stimuli with a Poisson distribution, while measuring the responses in intracellular recordings from motoneurons. This provides empirical data which validate and support the function of recurrent inhibition suggested by our previous biophysically-based model (Williams and Baker, 2009).

## Materials and Methods

The study was performed on four terminally-anesthetized female *Macaca mulatta* monkeys (monkeys Z, 5.5 kg, age 10 years; L, 6.1 kg, age 13 years; U, 10.5 kg, age 15 years; K, 10.7 kg, age 13 years). All experiments were performed under the authority of appropriate licenses issued by the United Kingdom Home Office, and were approved by the Animal Welfare and Ethical Review Board of Newcastle University.

### 

#### Anesthesia and surgical preparation

Animals were sedated with ketamine (10 mg/kg, i.m.), general anesthesia was induced by propofol (5–10 mg/kg) and then continued by inhalation of sevoflurane (2–4% in 100% O_2_) and intravenous infusion of alfentanil (10–15 µg/kg/h). The bladder was catheterized to allow urine drainage. An endotracheal tube was placed via a tracheotomy, and positive pressure ventilation began. Central arterial and venous cannulae were inserted via the neck vessels, to allow continuous blood pressure monitoring. Nerve cuff electrodes were placed in the left upper limb on the radial nerve in the axilla, the musculocutaneous nerve, the ulnar and median nerves in the arm, the deep radial nerve at the elbow, and the median and ulnar nerves at the wrist. A laminectomy was made to expose spinal segments C4-T2. Spinal clamps were placed on thoracic and lumbar vertebrae, and the head was mounted in a stereotaxic frame, angled to produce ∼60° neck flexion. Under an operating microscope, the spinal dura was opened and the dorsal roots severed, rootlet by rootlet, from C4-T2 to prevent afferent input from reaching the cord.

Once all surgical preparation was completed, anesthesia was switched to intravenous infusion of propofol (11–36 mg/kg/h) with alfentanil (10–32 µg/kg/h), since we have found that this yields better neural excitability than inhalational anesthetics such as sevoflurane. The respiratory gas supply was changed to a mixture of air and oxygen (final oxygen concentration ∼50%), to avoid potential problems with oxygen toxicity caused by breathing pure oxygen for long periods. The nerve cuffs were stimulated in turn (Model 2100 Isolated Stimulator, AM System Inc; biphasic pulses, 0.2 ms per phase), and the motor threshold determined. Subsequent stimulation used 3× motor threshold. Neuromuscular blockade was then initiated (atracurium, initial intravenous bolus 0.7 mg/kg followed by 0.7 mg/kg/h).

Anesthetic monitoring included heart rate, oxygen saturation, arterial and venous blood pressure, capnography, and core and peripheral temperature. The animal was warmed by a thermostatic heating blanket, and a supply of warmed air. Intravenous fluids were given (total fluid rate including drug infusions 5–10 ml/kg/h). Slowly rising trends in heart rate or blood pressure, or rapid changes following a noxious stimulus, were taken as evidence of waning anesthesia; supplemental drug doses were then given and/or infusion rates adjusted as appropriate.

#### Motoneuron recordings

The arachnoid was removed from the cord surface over the lateral funiculus, and a small patch of pia was removed. A pressure “foot” lightly pressed down over the patch, to reduce respiratory and cardiovascular pulsations. This foot was made of a loop of stiff silver wire, bent to shape; the wire was connected to an amplifier (Neurolog NL824 connected to NL820 Isolator, Digitimer Ltd, gain 5000, bandpass 1 Hz to 5 kHz), which provided epidural volley recording. Penetrations were made into the cord through the loop with glass micropipette electrodes (impedance 4–10 MΩ, filled with 2 m potassium acetate solution), driven with a piezoelectric micromanipulator (Burleigh PCS-6000, Thorlabs) and connected to a bridge amplifier (BA-03X, NPI; gain 20, bandpass DC-10 kHz). The location of the spinal surface was estimated from the change in potential on contact, although this can be subject to error because of fluid accumulation on the cord. The electrode was advanced while stimulating through one of the nerve cuff electrodes, chosen to produce the largest extracellular antidromic field in the microelectrode recording. Intracellular penetration into a motoneuron was indicated by a sudden drop in potential, often associated with spontaneous spiking. Motoneurons were identified by antidromic spikes following nerve stimulation, and were assigned to the following muscle groups based on the most distal nerve to which they responded: intrinsic hand (median or ulnar at wrist), forearm flexor (ulnar or median at arm, but not at wrist), forearm extensor (deep radial nerve), arm extensor (radial at axilla, but not deep radial). No motoneurons were recorded which responded antidromically to the musculocutaneous nerve. In addition, several cells were encountered which had all the properties of motoneurons based on our past experience [large cells, easily held stably in a penetration, broad spike, pronounced after-hyperpolarization (AHP)], but which did not respond antidromically to any of the implanted nerve cuffs. We classified these as unidentified motoneurons; they were usually recorded deep to motoneurons innervating wrist or elbow muscles, and are most likely to have innervated muscles acting around the shoulder and pectoral girdle because the most ventral parts of the ventral horn of the C7-T1 segments contain many motoneurons projecting to the latissimus dorsi and pectoral muscles (Holstege et al., 1987; Dasen et al., 2005).

After antidromic identification other nerves were tested in turn, and recordings assessed for synaptic responses. We then chose up to three nerves with candidate IPSPs detected online, and delivered further patterned combinations of stimuli to assess temporal properties of the responses. Stimuli to each nerve were timed as independent Poisson processes, with mean stimulus rate 10 Hz (Koželj and Baker, 2014). A stimulus was followed by a 3-ms period during which no further stimulus could be given. Such a Poisson process contains a broad range of spectral frequencies. Following completion of intracellular measurements, the electrode was withdrawn from the cell, and extracellular potentials at a nearby site recorded in response to the same nerve stimuli as tested intracellularly.

All signals, and digital markers indicating stimulus times, were sampled to computer hard disk (25 kSamples/s) using a 1401 laboratory interface and Spike2 software (both Cambridge Electronic Design).

At the end of the experiment, the animals were killed by overdose of anesthetic.

#### Measurements from experimental recordings

Off-line analysis used custom scripts written in the MATLAB environment (MathWorks). Averages were compiled of the intracellular records relative to stimulation of a given nerve. The amplitude of IPSPs was measured from onset to peak; any responses smaller than a threshold of 50 µV were discounted as unreliable. It remains possible that this criterion may have caused some connections to be missed, but we think this is unlikely to influence our results substantially. IPSPs recorded close to the resting membrane potential would be small, but sharp electrode intracellular recordings made *in vivo* inevitably lead to some leak currents and somatic depolarization away from the reversal potential for inhibitory synapses. Synapses from Renshaw cells are primarily on motoneuron dendrites; there is controversy in the literature on the number of synaptic contacts per cell: in mouse lumbar cord, a large number of synaptic contacts per cell often leads to large unitary IPSPs (Bhumbra et al., 2014), but a report in cat found an average of just three contacts between a single Renshaw cell and motoneuron (Fyffe, 1991), implying weaker connectivity. Nevertheless, the stimuli used here were strong (3× motor threshold), and would be expected to activate many motoneurons antidromically with subsequent activation of many Renshaw cells. These two factors would make IPSPs more pronounced, and hence less likely to be missed.

The AHP duration was estimated as the time after the spike when the membrane potential returned to half of its prespike level.

#### Calculation of coherence

We used spectral methods to define the temporal features of recurrent inhibition. Coherence and the coherence phase were calculated between the motoneuron membrane potential and the Poisson process stimulus times delivered to a single nerve, using methods similar to those previously described (Koželj and Baker, 2014). Briefly, the stimulus train was converted to a waveform sampled at 25 kHz, equal to one in the bin containing stimulus onset, and zero otherwise. This and the simultaneously-recorded motoneuron potential were divided into non-overlapping windows (length 2^14^ = 16,384 points), which were processed with fast Fourier transforms to yield coherence and phase (frequency resolution 1.53 Hz). When computing coherence with a stimulus, contamination by the stimulus artifact can have an important effect on the measurement of phase (Koželj and Baker, 2014). In the present recordings, the motoneuron membrane potential was corrupted not just by the stimulus artifact, but also by the small extracellular antidromic field potential elicited by the nerve stimulus and often visible in the intracellular recording. To reduce this contamination, for each recording and nerve we selected a poststimulus time window which encompassed both artifact and field. Following each stimulus, the membrane potential in this window was then replaced by a voltage linearly interpolated from the measurements on either side.

Coherence spectra were averaged across all available pairs of motoneuron and nerve stimulus. A significance limit for the averaged spectrum was determined according to the method used in Evans and Baker (2003). For each frequency bin, phase measurements from all individual spectra with coherence above significance were averaged using the circular mean (Fisher, 1993).

#### Characterization of non-linearities in recurrent inhibition

We wished to gain insight into how linearly IPSPs summed as a function of their temporal separation. This was achieved using conditional averaging applied to the experimental data. From the many available stimuli in the Poisson train, stimuli were selected which were preceded by an earlier stimulus in a narrow time window. These selected stimuli were used to compile an average of the motoneuron membrane potential. The average was corrected by subtraction of the expected response to the conditioning stimuli. The amplitude of the IPSP was measured and expressed as a percentage of the IPSP calculated from an average triggered by all stimuli. This procedure was repeated for conditioning windows over a range of times before the stimulus, allowing the construction of a conditioning curve.

#### Computational model

Experimental coherence results were compared with our previously-published computational model, which we have used to argue that recurrent inhibition acts to reduce physiological tremor around 10 Hz. The model is described in full in the original paper (Williams and Baker, 2009), which should be consulted for further details. Briefly, a pool of 177 motoneurons was simulated, each using a two-compartment conductance-based model. Each motoneuron received input from 20 Renshaw cells. To model the present experiment, each Renshaw cell received input from a separate group of 50 motoneurons. These were all activated synchronously by the motor nerve stimulation, which was timed as a Poisson process with the same parameters as in the experiment. The simulation was run for 1001 s; data from the first 1 s was discarded, to allow cell conductances to reach a steady state. Coherence and coherence phase were then calculated between the first motoneuron in the pool and the stimulus train.

#### Statistics

Recurrent inhibition was quantified as the amplitude (mean IPSP height, averaged across all motoneurons showing an effect), incidence (percentage of motoneurons with an IPSP), and the product of these two measures (amplitude × incidence). For display purposes, error bars were calculated on these values using a Monte Carlo resampling (bootstrapping) procedure to estimate 95% confidence intervals (Zaaimi et al., 2012). Values for a given class of motoneuron were compared with the overall distribution, also using a Monte Carlo approach, to test whether there were significant differences between motoneuron classes; *p* values were only accepted as significant if they passed the Benjamini–Hochberg procedure to adjust for multiple comparisons (Benjamini and Hochberg, 1995). This procedure does not provide a “corrected” *p* value, but only indicates whether a given comparison should be accepted; we prefer it over simpler approaches (e.g., Bonferroni correction) as it has improved power but preserves a false discovery rate of 5%. Accordingly, in the text we cite uncorrected *p* values, and indicate whether these were significant or not.

## Results

We report here intracellular recordings from 89 upper limb motoneurons in four monkeys, each tested for recurrent inhibition from multiple upper limb nerves, a total of 630 motoneuron-nerve pairs. Motoneurons were encountered between 2.1 and 4.2 mm below the estimated spinal cord surface.

**Figure 1.**
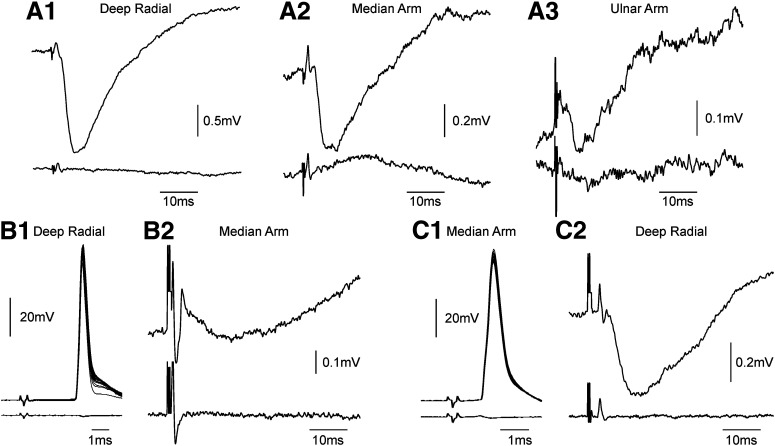
Example recordings. Top trace in each panel shows the intracellular recording from a motoneuron; bottom trace shows for comparison an extracellular recording made nearby. ***A***, An unidentified motoneuron. Recurrent IPSPs were visible from the deep radial (***A1***), median nerve at the arm (***A2***), and ulnar nerve at the arm (***A3***). ***B***, Motoneuron antidromically activated from deep radial nerve (***B1***). A recurrent IPSP was visible from the median nerve at the arm (***B2***). ***C***, Motoneuron antidromically activated from the median nerve at the arm (***C1***). A recurrent IPSP was visible from the deep radial nerve (***C2***). Traces are the average of all available stimuli, except for ***B1***, ***C1***, which present 20 overlaid single sweeps to show the consistency of the antidromic spike.

Figure 1 illustrates example recordings from three different motoneurons. In each case, a clear IPSP was visible in the intracellular recording, which was not present in an extracellular record to the same stimulus taken just after the electrode was removed from the cell (bottom trace in each pair). For the first motoneuron illustrated (Fig. 1*A*), there was substantial convergence, with IPSPs generated from the deep radial nerve, and the median and ulnar nerves at the arm. This motoneuron could not be antidromically identified from any of the implanted nerves. The second motoneuron was activated from the deep radial nerve (Fig. 1*B1*), indicating that it projected to forearm extensor muscles; this received a recurrent IPSP from forearm flexor motoneurons (Fig. 1*B2*). The third cell was activated from the median nerve at the arm (Fig. 1*C1*), this forearm flexor motoneuron received recurrent inhibition from motoneurons innervating forearm extensors (Fig. 1*C2*).

In our data, we did not see reliable short latency EPSPs following antidromic activation of motor axons as seen in the cat lumbar cord by Gogan et al. (1977) and rodent by Bhumbra and Beato (2018). A similar lack of direct motoneuron to motoneuron connections was also reported by Hahne et al. (1988) in the cat cervical cord. Longer latency facilitations following an IPSP (as reported by McCurdy and Hamm, 1994a) were sometimes seen in our recordings (Fig. 1*A*,*C*), but were not examined further.

### Distribution of recurrent inhibition

**Figure 2.**
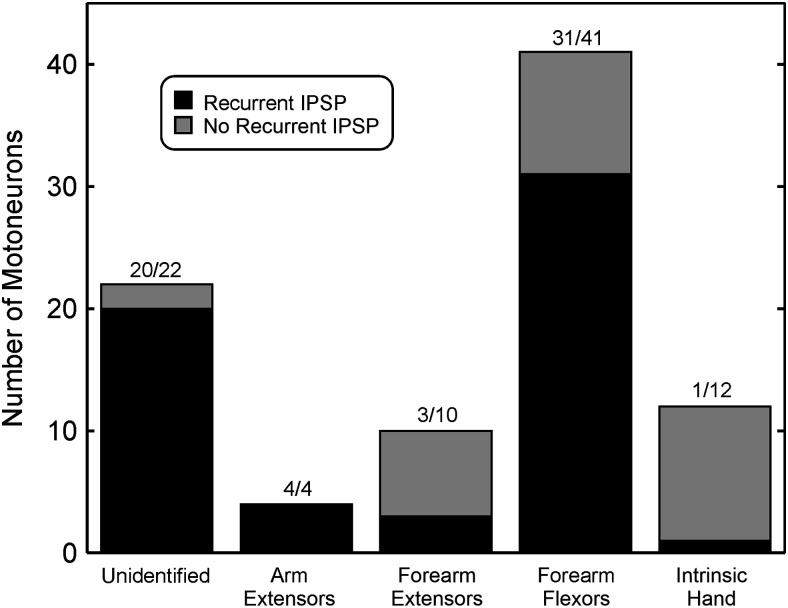
Sample size. Total bar height indicates the number of motoneurons of each class recorded. The bars are shaded to indicate the proportion with (black) or without (gray) recurrent IPSPs.

Figure 2 shows the distribution of our sample of motoneurons, classified according to which nerve could generate an antidromic spike. Bars are shaded to show the proportion of cells with (black) and without (gray) recurrent inhibition, evoked from any of the nerves stimulated. In many cases the presence of antidromic spikes prevented the detection of recurrent IPSPs evoked from other motoneurons activated by the same nerve. The proportions presented in Figure 2 are thus necessarily an underestimate. In addition, note that only four motoneurons projecting to arm extensors were identified, which is a very small sample. Overall, 59/89 (66%) of motoneurons showed recurrent inhibition. We tested the proportions found for the different motoneuron types against the null hypothesis that all had the same probability of showing IPSPs. Significantly fewer motoneurons projecting to intrinsic hand muscles had recurrent IPSPs than this overall proportion (proportion 8.3%, *p* = 0.0001, binomial test). IPSPs were seen in fewer motoneurons projecting in the deep radial nerve, and in more unidentified motoneurons, but these trends were not significant after correction for multiple comparisons (*p* = 0.042 and 0.017 respectively).

**Figure 3.**
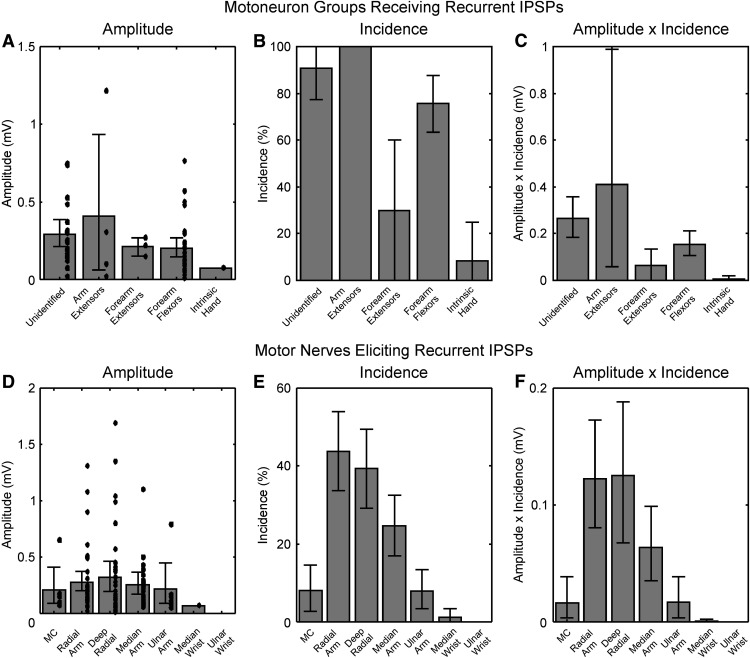
Measurements of recurrent inhibition. ***A***, Amplitude of recurrent IPSPs, measured as an average over all motoneurons where an IPSP was seen. ***B***, Incidence of recurrent IPSPs, equal to the percentage of motoneurons where an IPSP was seen. ***C***, The product of amplitude and incidence. Where a motoneuron received an IPSP from more than one nerve, the mean amplitude has been used in these calculations. ***D–F***, Similar plots to ***A–C***, but now classified by the stimulated motor nerve which elicited IPSPs, rather than the motoneuron which received them. In all plots, error bars show the 95% confidence limits around the measure, calculated using a Monte Carlo resampling procedure. Black dots in ***A***, ***D*** indicate individual amplitude measurements for single motoneurons (***A***) or nerves (***D***).

To quantify these effects further, we used three measures (Fig. 3), based on our previous work (Zaaimi et al., 2012). For each category, the IPSP amplitude was calculated as an average across all cells which showed an effect. The incidence measured the fraction of cells with an effect. Finally, we calculated the product of these two measures, incidence × amplitude, which provides a summary measure of effect. This is equivalent to calculating the mean amplitude, including as zeros motoneurons which had no IPSPs. Figure 3*A–C* shows the results of applying this approach to recurrent IPSPs in motoneurons projecting to different muscle groups. The general trend for mean IPSP amplitude (Fig. 3*A*) to be greater in the motoneurons in which recurrent IPSPs were most common (Fig. 3*B*) resulted in the of amplitude × incidence showing large differences (Fig. 3*C*). Recurrent inhibition was greatest in the motoneurons likely to project most proximally (unidentified motoneurons, which probably innervate muscles acting on the shoulder girdle, see Materials and Methods), to elbow extensors (radial nerve in the arm) or to wrist and digit flexors in the forearm (median nerve at the arm). Conversely, the amplitude of the single recurrent IPSP seen in an intrinsic hand muscle motoneuron was small. A Monte Carlo procedure was used to compare the amplitude × incidence values to those expected on the null hypothesis that they were similarly distributed across motoneuron categories. Recurrent inhibition was stronger for unidentified motoneurons, and weaker for intrinsic hand motoneurons, than expected by chance (*p* < 0.05 corrected for multiple comparisons).

Figure 3*D–F* shows the same approach applied to recurrent IPSPs evoked from different sources, in any motoneuron type. The nerves that evoked recurrent IPSPs of the largest mean amplitude (radial nerve in the axilla, deep radial nerve and median nerve in the arm; Fig. 3*D*) were also the nerves that most frequently evoked recurrent IPSPs (Fig. 3*E*). Stimulation of the ulnar nerve at the wrist failed to evoke recurrent IPSPs in any motoneurons, and the median nerve at the wrist evoked IPSPs in only one (which innervated forearm flexor muscles). Simulation of the musculocutaneous nerve and the ulnar nerve at the arm evoked quite large recurrent IPSPs, but in a relatively low proportion of motoneurons tested, suggesting a finer grained fractionation in the distribution of recurrent inhibition. Strikingly, stimulation of the radial nerve at the axilla and deep radial at the elbow evoked IPSPs in around 40% of the sampled motoneurons. This is a particularly high fraction, in the context of the rarity of recurrent IPSPs in some motoneuron types. Statistical comparison of the amplitude × incidence values indicated that the radial nerve in the arm and deep radial nerves generated significantly stronger recurrent inhibition than the whole population, whereas the musculocutaneous, ulnar nerve at the arm, and ulnar and median nerves at the wrist all generated significantly weaker recurrent inhibition (*p* < 0.05 corrected for multiple comparisons).

**Figure 4.**
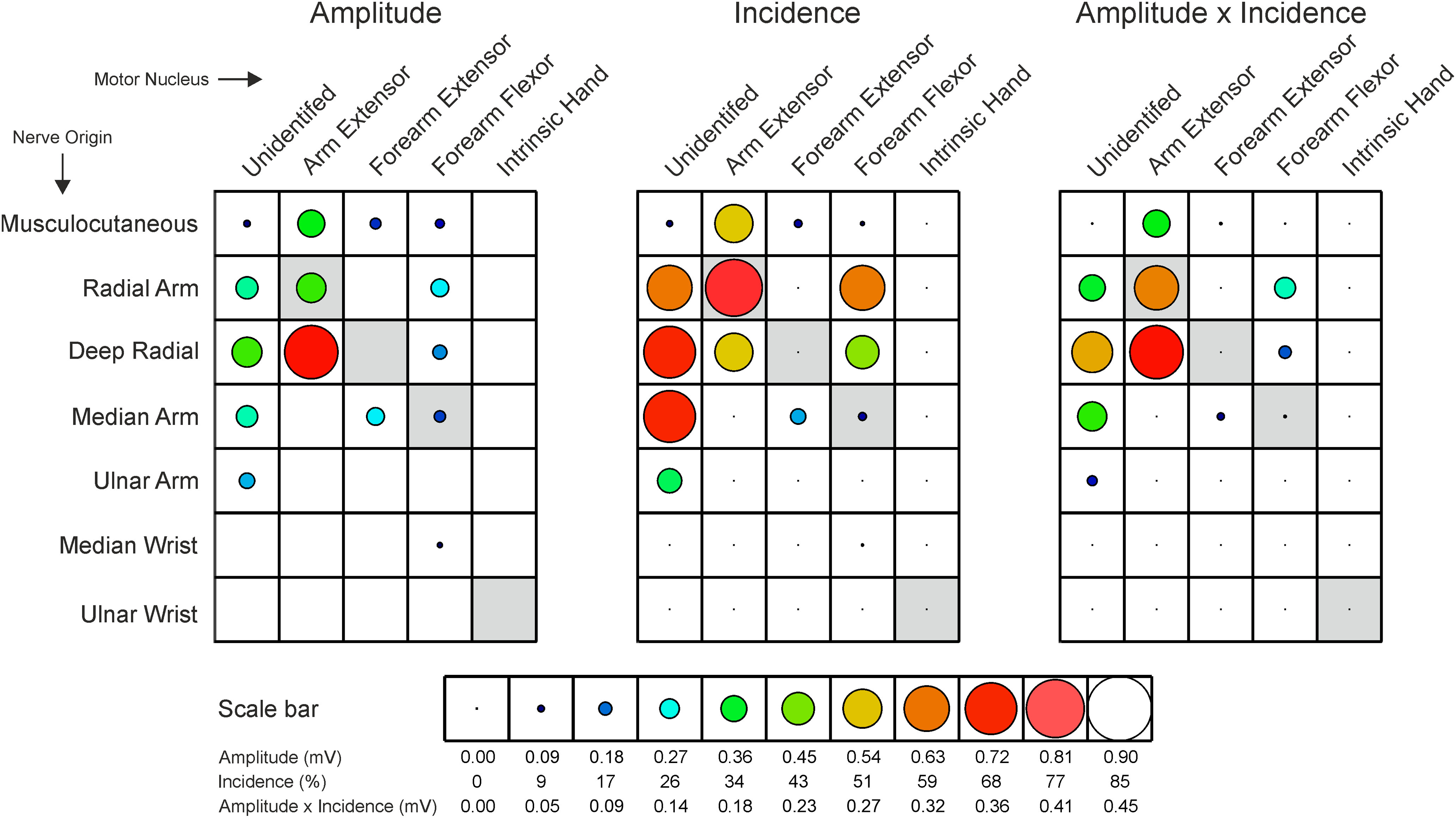
Distribution of recurrent inhibition. Plots show amplitude, incidence and amplitude × incidence of recurrent IPSPs for combinations of stimulated nerve and category of motoneuron. The diameter of the circles is proportional to the measure, on the scale shown at the bottom of the plot. Circles are colored according to the size of the measure, where the color is also indicated at the bottom. Gray boxes indicate combinations which relate to the homonymous nerve; in many cases, IPSPs could not be reliably measured in these cases because of the presence of an antidromic spike. The great majority of intrinsic hand motoneurons were activated from the ulnar nerve at the wrist (11/12), and of forearm flexor motoneurons from the median nerve at the arm (39/41); accordingly, the median wrist/intrinsic hand and ulnar arm/forearm flexor combinations can be considered reasonably reliable estimates, and have not been shaded gray.

The distribution of recurrent inhibition is explored further in Figure 4, which plots the amplitude, incidence and amplitude × incidence product for different input-output combinations. Importantly, this plot highlights with gray shading those combinations relating to homonymous connections. Detecting recurrent inhibition for homonymous connections is problematic, as the antidromic spike elicits an AHP which obscures the recurrent IPSP. In our recordings, we were only able to assess recurrent inhibition if the antidromic spike was blocked. The failure of the action potential to invade the soma then meant that no AHP was generated. Otherwise, measurements were not possible. Accordingly, we must take the values highlighted with gray boxes in Figure 4 as underestimates.

Some surprising and unexpected features emerge. First, the relatively large amplitude but infrequent recurrent IPSPs evoked by stimulation of the musculocutaneous nerve were evoked primarily in motoneurons antidromically activated from the radial nerve in the axilla, but not at the elbow, the great proportion of which are likely to be triceps motoneurons. Thus, triceps motoneurons, which are primarily elbow extensors, receive recurrent inhibition from their antagonist flexor muscles (the musculocutaneous nerve innervates the elbow flexors biceps and brachialis). A similar arrangement can be seen for forearm muscles: the deep radial nerve, which innervates wrist and long digit extensor muscles, frequently evoked recurrent IPSPs in forearm flexor motoneurons that innervate wrist and long digit flexor muscles, although these were generally smaller in amplitude than those in more proximal muscle motoneurons.

Second, Figure 4 reveals a high incidence of recurrent inhibition evoked from motoneurons that innervate muscles acting on one joint on motoneurons of muscles acting at other joints, including actions at some distance. Strikingly, motoneurons in the deep radial, ulnar and median nerves that innervate forearm muscles acting at the wrist and on the digits evoked larger and more frequent recurrent inhibition on the unidentified motoneurons that innervate shoulder girdle muscles. This pattern is substantially different to that seen in the cat.

Some features of the distribution do resemble the patterns previous reported in the cat: the intrinsic hand muscles rarely evoked recurrent IPSPs (one case, evoked in a forearm muscle innervated by the median nerve), and rarely received recurrent inhibition (one motoneuron, recurrent IPSPs from the deep radial and the radial nerve). This resembles the pattern seen in both cat hind and forelimb (Illert and Kümmel, 1999). Second, recurrent IPSPs, from any source, were much less common and were small in forearm extensor motoneurons, which has also been reported in the cat (Hahne et al., 1988) and motoneurons of the forearm wrist and digit extensor muscles in the cat were found to have few or no recurrent collaterals. While the receipt of recurrent IPSPs by deep radial motoneurons was low, resembling the findings in cats, the high frequency and strength of recurrent IPSPs evoked from the deep radial nerve differs from the cat (Hörner et al., 1991): in monkeys, these motoneurons seem generally to receive little recurrent inhibition, but to generate a lot, a pattern not found before.

### Relationship of recurrent IPSPs to the motoneuron AHP

A consistent finding in early reports in the cat was that the amplitudes of recurrent IPSPs are larger in motoneurons innervating slow twitch than fast twitch motor units (Eccles et al., 1961). This led to the suggestion that recurrent inhibition might play a role in the balanced recruitment of different motor units, although it has been pointed out that it is the effective current that is important, not the voltage response (Hultborn et al., 1988; Lindsay and Binder, 1991). To check whether recurrent inhibition in primate forelimb muscles might also show a relation between motor unit type and IPSP amplitude, we first measured the duration of the AHP. AHP duration is known to be correlated with the twitch time of the peripheral motor unit (Zengel et al., 1985; Bakels and Kernell, 1993; Gardiner, 1993; Gossen et al., 2003).

**Figure 5.**
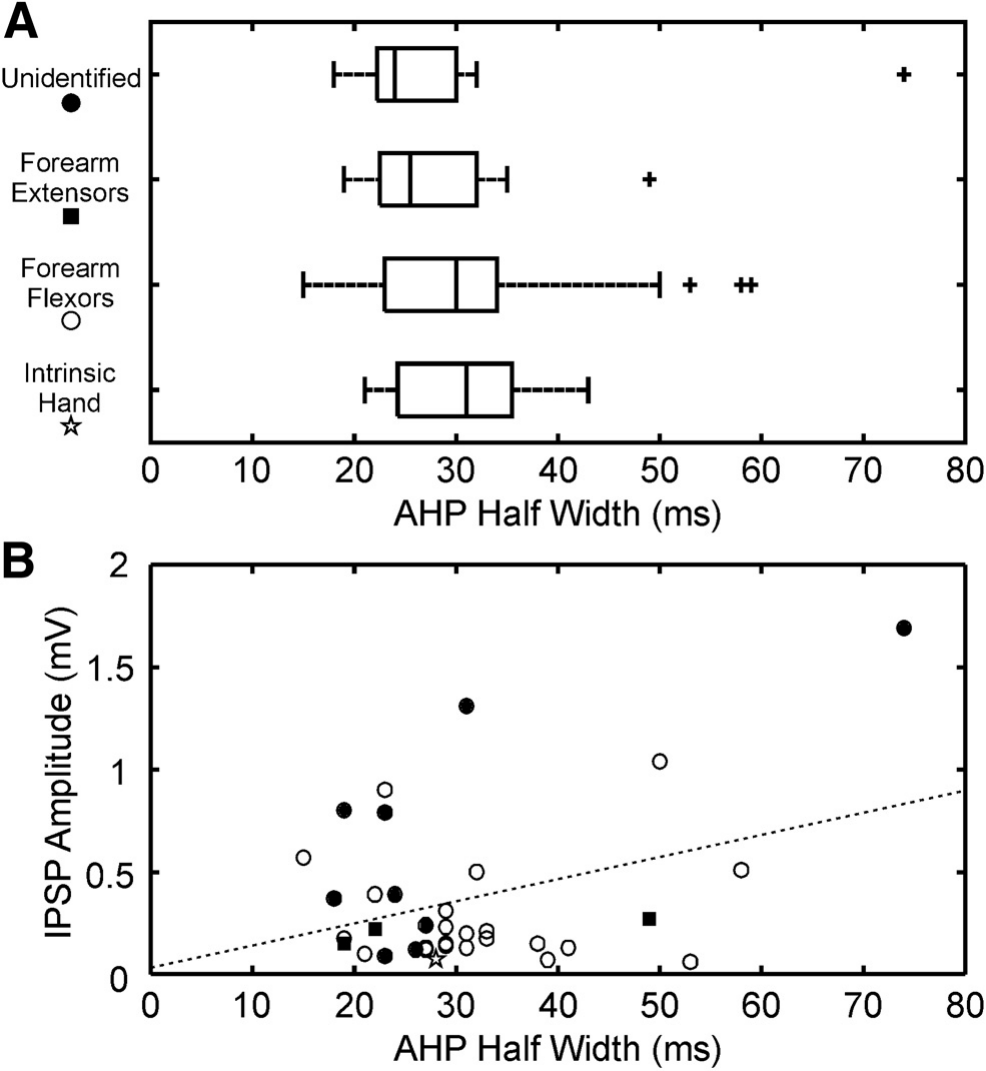
Motoneuron AHP. ***A***, Box-and-whisker plots showing the distribution of the AHP duration (measured as width at half maximum) for four classes of motoneuron. ***B***, Correlation of amplitude of recurrent IPSP with AHP duration. Motoneurons innervating different muscle categories are shown by the symbols identified on the ordinate of ***A***. Where a motoneuron received recurrent IPSPs from multiple nerves, the largest amplitude observed has been plotted. Dotted line shows significant linear correlation calculated using Pearson's correlation coefficient (*r*^2^ = 0.13, *p* = 0.034).

Figure 5*A* presents the distribution of AHP durations (measured as half-width: time to return to half maximum) for motoneurons in different categories. There was no significant difference between mean AHP durations between the four groups illustrated (ANOVA *F*_(3,56)_ = 0.29, *p* = 0.84), nor between their variances (Levene's test *F*_(3,56)_ = 0.71, *p* = 0.55). Using Pearson's correlation coefficient, there was a significant but weak relationship between AHP duration and amplitude of recurrent IPSPs (*r*^2^ = 0.13, *p* = 0.0342; Fig. 5*B*). However, this may be excessively influenced by large outlier values. Using the non-parametric Spearman's rank correlation coefficient (which has the disadvantage of less statistical power), the correlation was not significant (*r*^2^ = −0.035, *p* = 0.844).

### Temporal properties of recurrent inhibition

**Figure 6.**
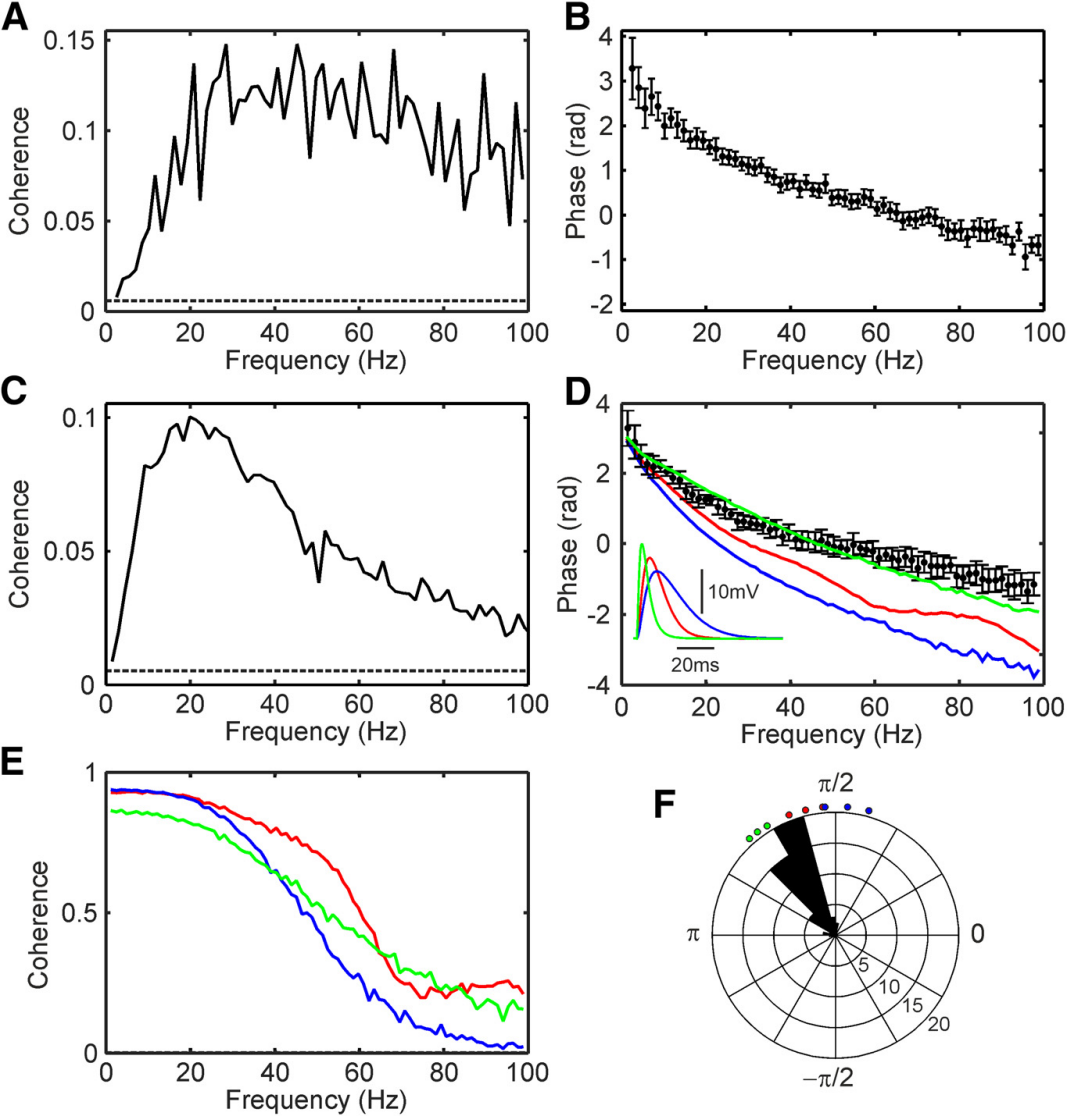
Coherence calculated for experimental data and a computational model. ***A***, Example coherence spectrum between Poisson stimulus train delivered to the deep radial nerve and the membrane potential of an unidentified motoneuron in monkey Z. ***B***, Phase for the coherence shown in ***A***. ***C***, Similar average coherence spectrum measured from 47 stimulus-motoneuron pairs. ***D***, Black points show the average coherence phase corresponding to the average experimental coherence in ***C***. Colored lines show coherence phase for three different simulations of a computational model, with different durations of EPSPs elicited in Renshaw cells by motoneuron axons [produced with conductance rise times of 1 ms (green), 4 ms (red), and 8 ms (blue), respectively; EPSPs shown in inset]. The modeled motoneuron was subjected to recurrent inputs from a motor axon, stimulated with a Poisson train, as in the experimental condition. ***E***, Similar coherence spectra for the computational models, corresponding to the phase shown in ***D***. ***F***, Circular histogram of coherence phase in the 8- to 12-Hz band, for the 47 experimental stimulus-motoneuron pairs illustrated as averaged coherence and phase in ***C***, ***D***. Each bin in this frequency range with coherence significantly different from zero has contributed one count to the histogram. Dots outside the circular histogram indicate the phase measured from the three simulations illustrated in ***D***, ***E***, for the three frequency bins in the 8- to 12-Hz range. Dotted lines in ***A***, ***C***, ***E*** indicate significance level for coherence spectra (*p* < 0.05). Error bars in ***B***, ***D*** indicate 95% confidence limits on phase.

Coherence analysis was used to determine how effectively recurrent inhibition transferred information at different frequencies back to the motoneuron. Figure 6*A* shows coherence calculated between a single example unidentified motoneuron and stimuli given to the radial nerve at the arm. Coherence was small at low frequencies, but rapidly rose to a peak at ∼30 Hz, then gradually declined, remaining clearly above significance even up to 100 Hz; it was significantly different from zero for all frequencies shown (significance limit indicated by dotted line). The coherence phase (Fig. 6*B*) was high at the lowest frequencies, fell steeply until around 20 Hz, after which the phase-frequency relationship was approximately linear. A linear phase-frequency relationship is compatible with a simple delay; the slope can provide an estimate of this delay (Witham et al., 2010). Here, the measured slope corresponded to a delay of 4.3 ms (95% confidence limit 4.1–4.6 ms).

Figure 6*C* presents the average coherence spectrum, computed over 47 cell-nerve pairs. The averaged coherence was low at low frequencies, rose to peak at 20 Hz, and then gradually declined at higher frequencies, although it remained significantly different from zero up to 100 Hz. The average phase (Fig. 6*D*, black circles) showed a similar profile to the example cell of Figure 6*B*.

We have previously used a computational model to investigate how recurrent inhibition could modify oscillations (and thereby synchrony) in motoneurons (Williams and Baker, 2009). To provide a direct comparison of our model with the experimental data, we simulated in the model a situation in which Renshaw cells were stimulated synchronously by motor nerve activation timed as a Poisson train. This allowed the simulated and experimental data presented here to be subject to the same analysis. As in the original model, we simulated three different situations, with EPSPs generated in Renshaw cells by motoneurons of different duration. Figure 6*E* presents the coherence spectrum between the stimulus train and motoneuron membrane potential for these simulations. The different colored traces show results for different EPSP conductance rise times (green, 1 ms; red, 4 ms; blue, 8 ms). These are the same rise times and plotted with the same color code as used in Williams and Baker (2009; their Fig. 4C–H). The different simulations gave broadly similar coherence spectra, although as expected the simulation with widest EPSP showed reduced coherence at higher frequencies. Like the experimental measurement in Figure 6*C*, the coherence for the simulated data declined above 20 Hz. However, in the simulation coherence remained high for frequencies below 20 Hz, whereas in our experimental measurements, coherence fell below 20 Hz.

The coherence phase calculated from these simulations is shown in Figure 6*D*, superimposed on the phase estimated from the experimental data. The model with the briefest Renshaw cell EPSP (green line) matched the experimental data most closely.

In our previous paper using this model, we concluded that recurrent inhibition acted to reduce low-frequency motoneuron firing synchrony contributing to physiological tremor in the 8- to 12-Hz band. The phase behavior is thus of especial interest in this frequency range. Figure 6*F* accordingly presents a circular histogram of the coherence phase values measured from the 47 experimentally-recorded motoneuron-nerve pairs between 8 and 12 Hz. The phases were tightly clustered, with a mean of 2.12 radians. This compared with 1.48, 1.80, and 2.21 radians for the same phase measurements from the simulations with brief, medium, and long duration EPSPs, respectively (phase measures from individual frequency bins shown as colored dots outside the rose plot of Fig. 6*F*). Our experimental data thus match the computational model with the shortest duration EPSPs very well.

**Figure 7.**
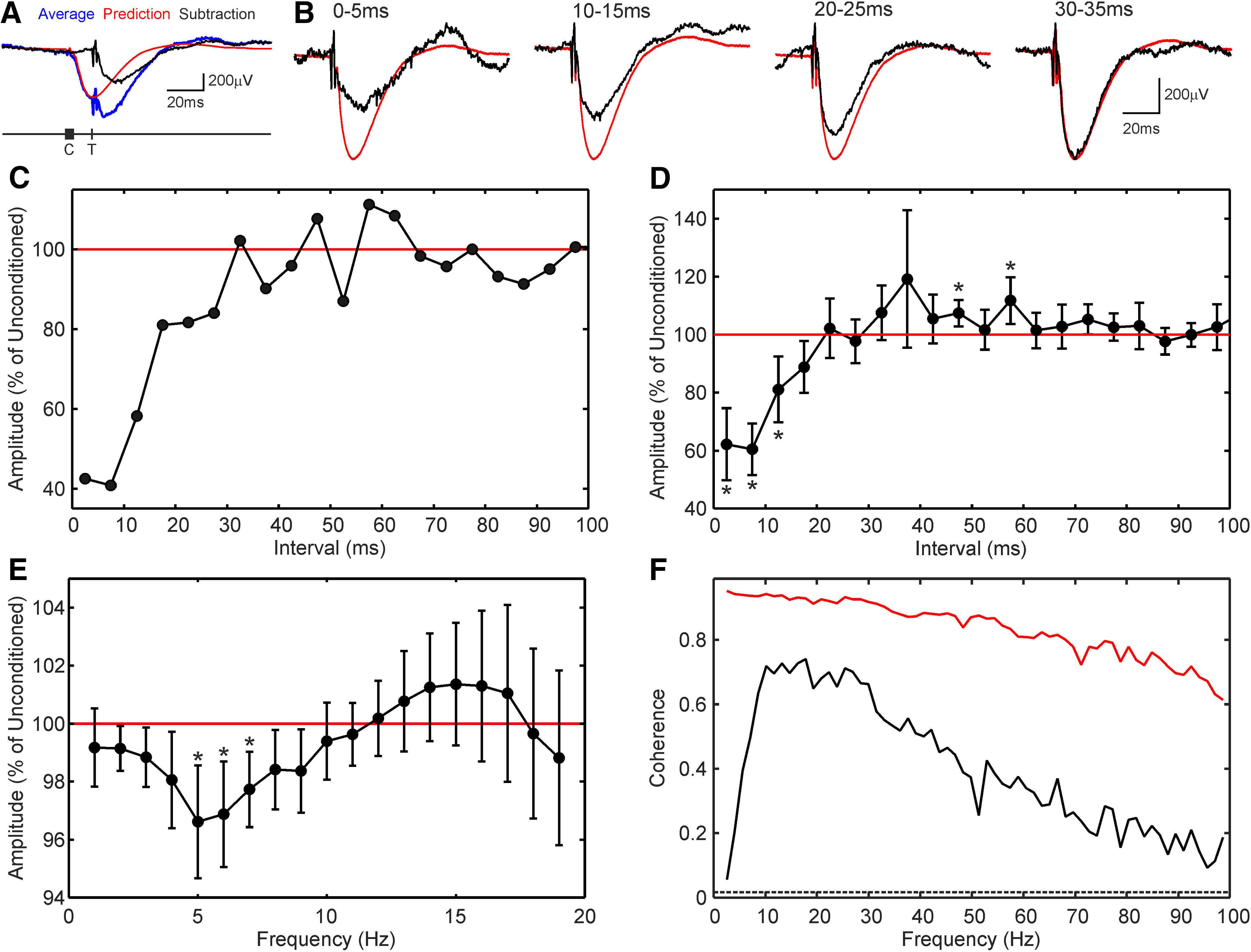
Non-linearities in recurrent inhibition. ***A***, Selective averages of motoneuron membrane potential were compiled (blue), triggered by a stimulus (at time label T) when it was preceded by a conditioning stimulus within a 5-ms window (time window label C). A prediction of the expected response to the conditioning stimuli if delivered alone was generated (red), and subtracted from the averaged response to C plus T stimuli to give the response to the triggering stimulus T alone (black). ***B***, Each panel shows the results of such an analysis, for a different conditioning stimulus window. Black traces show the subtracted average; red shows the average from all available stimuli, without selection, for comparison. ***C***, Amplitude of IPSP measured from motoneuron-nerve pair illustrated in ***A***, ***B***, as a function of the interval between the two stimuli. Amplitude is plotted as a percentage of that measured from the all-stimulus average (red line, 100%). ***D***, Average of conditioning curves similar to that in ***C***, across 14 stimulus-motoneuron pairs with sufficient signal: noise ratio for reliable IPSP amplitude measurement. Error bars show 95% confidence limits (2xSEM). ***E***, Similar curve as ***D***, but compiled for longer interstimulus intervals, with 100-ms-wide conditioning test windows. Abscissa is expressed as frequency, the reciprocal of the interstimulus interval. Asterisks in ***D***, ***E*** mark points significantly different from 100% (*p* < 0.05, corrected for multiple comparisons). ***F***, Black trace shows the experimentally-determined coherence spectrum between the stimulus train and motoneuron potential for the cell illustrated in ***A–C***. Red trace shows the coherence calculated between the stimulus train and the convolution of the unconditioned IPSP (red traces in ***B***) with the stimuli. This is the coherence spectrum expected if transmission was linear.

Coherence is a linear analytical technique. Several known features of the recurrent inhibition circuit would be expected to introduce non-linearities; examples would be the spiking threshold of the Renshaw cells and shunting inhibition at the motoneuron membrane. Paired-pulse facilitation or depression between successive activations of a synapse represent another form of non-linearity. Non-linear summation of IPSPs was investigated in our experimental dataset by constructing conditional averages; the basis of this analysis is illustrated in Figure 7*A*. We first generated an average of motoneuron membrane potential, triggered by stimuli which have been selected to have a preceding stimulus in a 5-ms window (Fig. 7*A*, blue trace; labels T and C indicate the triggering and conditioning stimuli, respectively). A prediction of the response to the conditioning stimuli was then generated, based on the average compiled from all stimuli, suitably time shifted (Fig. 7*A*, red trace). This was subtracted from the conditional average, yielding the measured response to the second stimulus (Fig. 7*A*, black trace).

Figure 7*B* shows example corrected conditional averages compiled in this way, for different conditioning windows as illustrated (black traces). Overlain is the average formed from all stimuli (red line) for comparison. If IPSPs sum linearly, these traces should be identical. It is apparent that this was not the case; the IPSP produced by a stimulus which occurred 0–5 ms after a preceding stimulus was greatly reduced, and this paired-pulse suppression persisted even for intervals of 20–25 ms. Figure 7*C* plots the IPSP amplitude as a function of the interstimulus interval. For this cell, the IPSP only recovered to the level seen in the unconditioned average for intervals around 30 ms.

Figure 7*D* shows a similar conditioning curve, averaged over 14 motoneuron-nerve pairs where the IPSPs were sufficiently clear to be measured following the greatly reduced number of triggering stimuli available in a conditioned average. The IPSP was significantly reduced for intervals up to 15 ms.

Further investigation revealed that nonlinear IPSP summation also occurred at much longer intervals (Fig. 7*E*). To compile this plot, conditioning windows 100 ms wide have been used, with the center of the window placed 53–1000 ms before the triggering stimulus. To improve visualization, the abscissa has been plotted as frequency (the reciprocal of interstimulus interval). IPSPs were significantly reduced if they occurred at rates between 5–7 Hz.

Non-linearities in IPSP summation will therefore reduce the ability of recurrent inhibition to transmit information at both high (Fig. 7*D*) and low (Fig. 7*E*) frequencies. To illustrate the size of this effect, we took the IPSP estimated using an average of all stimuli for the cell illustrated in Figure 7*B*, red line, and convolved it with the Poisson train used to determine the stimulus timing. This provided a simulation of the membrane potential, as it would have appeared if IPSPs had summed linearly. The coherence between this signal and the stimulus is illustrated in Figure 7*F*, red line, overlain on the actual coherence seen between this motoneuron membrane potential and stimulation (Fig. 7*F*, black line). The experimentally-measured coherence was lower than for the simulated signal at all frequencies, presumably reflecting membrane noise in the motoneuron which was unrelated to the stimulus. However, the curves diverge to a much greater extent at high and lower frequencies, with the highest coherence in the 10- to 30-Hz region.

## Discussion

### Distribution of recurrent inhibition

**Figure 8.**
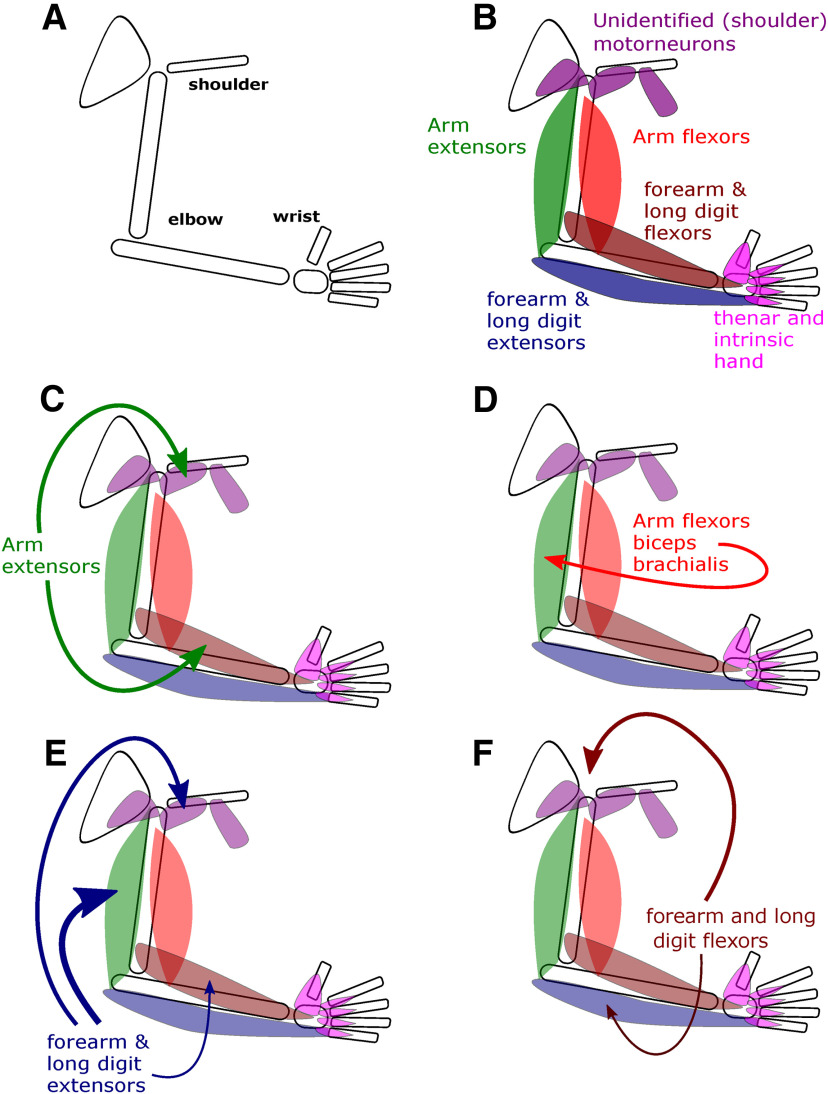
Schematic diagram representing the distribution of recurrent inhibition found in this study. Muscles are shown schematically and color coded by blocks innervated by the nerves tested. By exclusion (not antidromically activated by branches of the radial, median, ulnar, or musculocutaneous nerve) unidentified motoneurons are considered to innervate shoulder or pectoral girdle muscles (shown in purple). Arrows indicate the connections found, line thickness indicating strength (amplitude × incidence). ***A***, ***B***, Skeletal elements of the upper limb and the major muscle blocks tested. ***C***, Recurrent inhibition from arm extensors was distributed to forearm (wrist and long digit) flexors and shoulder muscles. ***D***, Recurrent inhibition from elbow flexors to elbow extensors. ***E***, Recurrent inhibition from forearm (wrist and long digit) extensors to elbow extensors, shoulder muscles, and more weakly to forearm flexors. ***F***, Recurrent inhibition from wrist and long digit flexors to shoulder muscles and more weakly to wrist and long digit extensors. Because homonymous connections could not be identified for most motoneurons, they are excluded. Thenar and intrinsic hand muscles neither generated nor received recurrent inhibition.

This study provides the first direct observation of recurrent inhibition in primate upper limb. Our findings confirm previous indirect observations in human (Katz et al., 1991; Katz and Pierrot-Deseilligny, 1999) and direct observations in cat (Hahne et al., 1988) but also contain some substantial differences in distribution. Figure 8 presents, in schematic form, our major findings.

Recurrent inhibition is not ubiquitous; in our data, it was rarely evoked from or received by motoneurons of intrinsic hand muscles (Figs. 2–4). This resembles the cat hindlimb, where distally-projecting motoneurons are generally devoid of recurrent collaterals (Cullheim and Kellerth, 1978). Earlier studies in cat forelimb stimulated the ulnar and median nerves only at the elbow, and hence could not resolve whether any recurrent inhibition elicited arose from motoneurons projecting to intrinsic hand muscles or forearm flexors (Hahne et al., 1988). Previous indirect studies in man failed to find recurrent inhibition in intrinsic hand muscles (Katz and Pierrot-Deseilligny, 1999), although this has been questioned (Piotrkiewicz and Młoźniak, 2015).

There were two striking features in our data. First, recurrent inhibition was seen between muscles classically viewed as antagonists: from elbow flexors to extensors and between the forearm wrist and digit flexors and extensors (Figs. 4, 8*D–F*). Second, recurrent inhibition frequently occurred across joints. Particularly noticeable were the large effects in unidentified motoneurons, situated deep to antidromically-activated cells. This location contains motoneurons projecting to proximal muscles including latissimus dorsi, pectoralis major and minor (Sterling and Kuypers, 1967; Hörner and Kümmel, 1993) which move and stabilize the shoulder joint and scapula. Finding that these muscles received frequent large recurrent IPSPs evoked by stimulation of forearm flexor and extensor muscle nerves (Fig. 8*E*,*F*) was surprising.

General organizational principles of recurrent inhibition arose from extensive studies in cat hindlimb (Eccles et al., 1961; Hultborn and Pierrot-Deseilligny, 1979); results in cat forelimb are similar (Hahne et al., 1988). Stimulation of a muscle nerve evokes recurrent inhibition primarily in motoneurons of the same muscle and its synergists (Eccles et al., 1961). The distribution generally follows the functional flexor/extensor organization of the stretch reflex. This organization integrates Renshaw cells into the motor output stage, allowing control of output gain (Hultborn et al., 1979). Recurrent inhibition in cat hindlimb generally fits this pattern, possibly because muscle synergies during locomotion broadly fit flexor/extensor categories. Where there are known exceptions, recurrent inhibition appears organized according to functional synergies, rather than stretch reflex connectivity (e.g., for flexor digitorum longus and flexor halucis longus; Hamm, 1990). Similarly, motoneurons innervated by the deep radial nerve in the cat forelimb both receive and generate weak or no recurrent inhibition (Hahne et al., 1988; Hörner et al., 1991). These anatomically adjacent muscles contribute to different synergies during locomotion (Krouchev et al., 2006).

In the present study, recurrent IPSPs were sparse in deep radial motoneurons, as in the cat. However, these motoneurons generated substantial and frequent recurrent IPSPs in other motoneurons (Figs. 4, 8*E*). We did not identify the specific muscles innervated further, so we cannot provide further fine-grained information. However, given the functional fractionation of actions in cats, the flexibility in how these muscles are used suggests an even more fractionated action in primates. Studies in human have suggested that muscles acting about the wrist may have a different organization of spinal interneurons, reflecting their ability to reconfigure agonist/antagonist coupling for both flexion/extension and ulnar/radial deviation movements (Aymard et al., 1995; Wargon et al., 2006). Additionally, because the tendons of forearm finger flexor muscles traverse the wrist, activation of these muscles alone will generate an unwanted wrist flexion; only by co-activating wrist extensors can isolated finger flexion be generated. These biomechanical constraints may also be responsible for the complex organization of recurrent inhibition which we observed.

The very sparse recurrent inhibition in intrinsic hand muscles and wrist and digit extensors fits with the general notion that recurrent inhibition is weaker in muscles dominated to a greater extent by motor cortical control (Fritz et al., 1985). The wrist and digit extensors are particularly profoundly impacted by cortical lesions (Kamper et al., 2003), and their neural control shows a reduced ability for plasticity (Godfrey et al., 2013; Foysal and Baker, 2019). The present results support the idea that these muscles may have fundamental differences in their neural control compared with the forearm flexors.

### Temporal filtering by recurrent inhibition

Several authors suggested that recurrent inhibition limits synchrony between motoneurons, thereby reducing physiological tremor (Adam et al., 1978; Windhorst et al., 1987; Windhorst and Kokkoroyiannis, 1992; Maltenfort et al., 1998). Others suggested the opposite, that common Renshaw cell inhibitory feedback enhances motoneuron synchrony (Elble and Randall, 1976; Davey et al., 1993; Mattei et al., 2003; Uchiyama and Windhorst, 2007). Our previous computational model attempted to reconcile these views (Williams and Baker, 2009). We suggested that recurrent inhibitory feedback would cancel synchronous oscillations around 10 Hz but augment them at 30 Hz. For most muscle groups, this would be beneficial as physiological tremor is typically in the 8- to 12-Hz range, whereas 30-Hz oscillations are beyond the low-pass filter imposed by the sluggish dynamics of motor unit twitch times and limb inertia. However, the digits' small size gives them an unusually high mechanical resonant frequency close to 30 Hz (Elble and Koller, 1990). Enhancing 30-Hz drive to muscles acting on the fingers could therefore produce deleterious fine tremor. This may explain why recurrent inhibition is not found in these motor nuclei, a finding which we have confirmed for primates in the present work.

Our previous computational model was constructed using a range of reasonable values for parameters gleaned from recordings in cat. The present experiments have, for the first time, made direct phase-frequency measurements of the recurrent inhibitory circuit in primate upper limb motoneurons. Experimentally-measured coherence phases agreed closely with simulation (Fig. 6*D*,*F*). The best agreement was seen using the shortest duration EPSPs in Renshaw cells (Fig. 6*D*,*F*, green traces). In our previous work, tremor was reduced by 40%, 58%, and 72% using EPSPs with same fast, medium, and slow rise times as tested here. Our results therefore imply that tremor reduction is at the lower end of previous estimates.

Importantly, note that the phase-frequency measurements made here cannot be used directly to infer effects on physiological tremor. Our protocol involved measuring motoneuron responses to strong stimulation of a whole motor nerve, causing synchronous activation of many motor axons. The motoneurons were at rest, so that synaptic inputs did not interact with intrinsic cell properties seen during repetitive firing such as the AHP. Instead, our approach was to simulate the same artificial situation as we had tested experimentally, and to confirm that the model yielded similar results. With the model thus validated, we can then have high confidence in our previous results which simulated the more natural situation of an active motoneuron pool responding to descending inputs.

Previous work in cat used Poisson train stimulation of motor axons (Christakos et al., 1987) and found that frequencies up to 50–100 Hz were reliably represented in the Renshaw cell responses. Our work additionally includes the next stage in the recurrent inhibitory pathway from Renshaw cells to motoneurons. We also found significant coherence up to 100 Hz (Fig. 6*C*). The same group also explored non-linearities in Renshaw cell responses (Windhorst et al., 1987; Laouris et al., 1988). Again similar to our findings (Fig. 7), these authors reported a paired-pulse depression of responses, indicating that much of the non-linearity at short interstimulus intervals which we observed (Fig. 7*D*) occurs at the level of the Renshaw cell. Paired pulse depression at short intervals led to reduced coherence above 30 Hz between the motoneuron membrane potential and stimulus train (Fig. 7*F*). This is however unlikely to have consequences for motor control, because slow motor unit twitch times already severely attenuate such high frequencies in the final muscle output (Mannard and Stein, 1973).

We also observed a nonlinear reduction in recurrent inhibition at frequencies <10 Hz (Fig. 7*E*), which substantially reduced coherence at these frequencies (Fig. 7*F*). Our computational model did not incorporate this effect so that, unlike the experimental measurements, coherence in the model remained high at low frequencies (Fig. 6*E*). It remains unclear what causes this low-frequency non-linearity. One possibility is that Renshaw cells inhibit other Renshaw cells (McCurdy and Hamm, 1994b). This leads to a late recurrent facilitation in motoneurons, which was visible in some of our records (Fig. 1*A2*), and could plausibly generate nonlinear interactions at long intervals. However, coherence between motor axon stimulation and Renshaw cells does not fall below 10 Hz (Christakos et al., 1987), suggesting that the non-linearity arises at the motoneuron. Motoneuron input-output properties contain substantial non-linearities (Binder et al., 2020). One candidate mechanism could be the delayed inward current *I_h_*, which is activated by hyperpolarization and found in adult motoneurons (Milligan et al., 2006; Howells et al., 2012). If activated by a recurrent IPSP, an *I_h_* current could reduce the amplitude of a subsequent IPSP in a time window possibly compatible with our observations. The motoneuron model of Powers (1993), which formed the basis of our model does not include *I_h_*, possibly explaining why our simulations did not show reduced low-frequency coherence. Frequencies below 10 Hz contain most of the voluntary motor command (Negro et al., 2009; Susilaradeya et al., 2019). Limiting recurrent inhibition in this band may have functional utility, by reducing the cancellation of input modulations generated by descending commands to achieve a motor goal.
